# A Crisis in the Health System and Quality of Healthcare in Economically Developed Countries

**DOI:** 10.3390/ijerph20010469

**Published:** 2022-12-28

**Authors:** Magdalena Biel, Katarzyna Grondys, Ane-Mari Androniceanu

**Affiliations:** 1 Faculty Management, Czestochowa University of Technology, Armii Krajowej 19b, 42-200 Czestochowa, Poland; 2 Doctoral School of Management, The Bucharest University of Economic Studies, Piața Romană 6, 010374 Bucharest, Romania

**Keywords:** healthcare, quality of service, crisis in health system, management of resources

## Abstract

A health crisis caused by a pandemic tested the effectiveness of national healthcare systems by testing both financing and organizational and technical performance of patient care. At that time, the structural flaws in healthcare systems and inequalities in the level of healthcare in its different dimensions and countries due to resource constraints were highlighted. Therefore, the paper concentrates on investigating how the crisis in the health system affects the quality of healthcare services as a result of changes in the availability of financial, material, and human resources belonging to this system. The quantitative data, in terms of healthcare characterizing the OECD countries and selected non-member economies, treated as an example of economically developed regions, were chosen for the analysis. The study included five areas of resources, i.e., demographic, financial, human, technical, and the delivery of basic services in healthcare. T-test method for dependent samples, supplemented with Hedge’s g statistics, was applied to test the differences between the mean values of individual indicators. The results indicate the occurrence of changes in some areas of the healthcare system due to a crisis. Identifying areas that are particularly vulnerable to sudden changes in the healthcare system helps to understand which resource areas need to be strategically managed first, as shifts in levels respond to deteriorating healthcare quality outcomes.

## 1. Introduction

Healthcare is an object of great interest since it is the world’s most rapidly developing service sector [1]. Therefore, concerns are growing about the quality of healthcare and patient safety, especially in terms of costs, malpractice, or healthcare reform [2]. The research into the quality of healthcare services is significant since it relates to one of the most important sectors, which directly affects the lives of individuals and society [3]. Additionally, one may observe the gap in healthcare access and quality between the most and least disadvantaged groups [4]. The COVID-19 pandemic worsened health inequalities even further [5,6]. According to the authors of the report “2022 Global Health Care Outlook. Are we finally seeing the long-promised transformation?” [7], prepared by the Deloitte consulting company, disproportions are nothing new, but a crisis caused by the pandemic influenced social groups that commonly experienced barriers to effective care. At that time, the structural flaws in healthcare systems and inequalities in the so-called social determinants of the level of healthcare were highlighted. Undoubtedly, in this situation, for the survival of organizations operating in the sector, the key business strategy is to satisfy customer needs and expectations [8]. Due to the private and public deliverers of medical services [9], competition in the sector increased, which forced healthcare organizations to improve quality and overcome shortcomings [10]. It is one of the key sectors since it also affects other areas of operation of specific countries, such as business, politics, society, or finance [11,12]. Therefore, it is essential to understand the importance of the quality of healthcare not only at the micro level, concerning individual healthcare facilities, but also at the macro level, from the point of view of the entire healthcare system. Depending on the country, this system may be organized differently and be based on public or private resources or a combination of them. Despite ownership differences, both public and private entities, thanks to contractual relations, may belong to one integrated health system. The general framework of this system and the scope of its integration are usually determined by government regulations and affect the efficiency and responsiveness of healthcare. Financing health systems in most countries is based on public, private, and external sources [13]. Unfortunately, it is often insufficient, leading to underfunding of public healthcare in some countries [14]. Increasing costs and concern for the quality of health services make many countries face the issue of health system reform and the appropriate balance of public and private medical services [15]. Therefore, the high quality of the functioning of the entire system and its health services is of key importance and can be the answer to many emerging problems.

The quality of healthcare services is much more difficult to define and measure than in other sectors [16]. Different characteristics of the healthcare sector, such as immateriality, heterogeneity, and simultaneity, make it difficult to define and measure quality [17,18,19]. Fundamentally, however, the quality of healthcare services depends on the service process and interaction between the customer and the service provider. Some characteristics of the healthcare quality, such as timeliness, consistency and accuracy, are difficult to measure beyond the subjective judgement of the customer. Healthcare services may differ between their providers, recipients, or places of delivery, and quality standards are difficult to establish. They are manufactured and consumed simultaneously, which impedes their quality control. The patient cannot assess the “quality” before purchasing the service and its consumption. Therefore, healthcare outcomes cannot be guaranteed, and their quality level is subjective, complex, and multidimensional [20]. While making any efforts to define, measure and improve the quality of healthcare, one should consider various perspectives, desires, and priorities of healthcare stakeholders. 

Although much empirical research was conducted to assess the quality of healthcare organization, little research was carried out to identify the impact of the crisis in healthcare on the quality of healthcare services. Most research was limited to identifying factors affecting the quality of medical services, patient satisfaction or multi-faceted assessment of the quality of medical services from the point of view of service providers and recipients. In addition, most studies were limited to single hospitals/groups of hospitals or were based on the analysis of existing literature. For example, Khamis and Njau [21] determined patients’ level of satisfaction with the quality of healthcare delivered at the outpatient department in selected hospitals. Abuosi and Atinga [22], based on questionnaires administered to 250 patients, examined patients’ hospital service quality perceptions and expectations using SERVQUAL. Ghahramanian et al. [23] investigated the quality of healthcare services from patients’ perspectives and its relationship with patient safety culture and nurse–physician professional communication. They conducted research on a group of 300 surgery patients and 101 nurses caring for them in a public hospital in Tabriz–Iran. Mohebifar et al. [24] examined 360 patients from six academic hospitals in Qazvin and focused on evaluating the quality of service in teaching hospitals using an importance–performance analysis matrix. Kitapci, Akdogan, and Dortyol [25] conducted research on the effect of satisfaction on word-of-mouth communication and repurchase intention and searched for a significant relationship between these factors. Their study included 369 patients facing a range of services. Hincapie et al. [26] evaluated the association between patients’ perceived healthcare quality and self-reported medical, medication, and laboratory errors in a multinational sample. They based their research on a CWF survey, which was conducted in 11 countries in 2010 and consisted of a national representative sample of adults 18 years and older. Whereas Padma, Rajendran, and Sai [27], based on the existing models and the literature on healthcare services, determined the dimensions of service quality in Indian hospitals from the perspectives of patients and their family members/friends. Naidu [28], using a systematic review of 24 articles from international journals, tried to build a comprehensive conceptual model to understand and measure variables affecting patient satisfaction-based healthcare quality.

In times of ubiquitous changes and economic crisis, special attention should also be paid to resources and facilities, which constitute important environmental factors that affect providing quality healthcare services [20]. This was particularly evident during the COVID-19 pandemic, which intensified the existing problems in healthcare. It can even be said that it significantly changed the entire system and its services [29,30]. It turned out to be a huge burden for the healthcare system, causing the need to adapt and concentrate virtually all resources on one threat, while limiting the availability of diagnostics and treatment in other areas [31,32]. Therefore, national governments decided to implement special solutions and strategies to prevent the spread of the virus and support healthcare [33]. Various restrictions were imposed on entire societies, promoting self-isolation and maintaining social distance [34]. In many countries, healthcare systems were temporarily and drastically redesigned. Specialist hospitals and wards for COVID-19 patients (including temporary ones) were established, to which some healthcare professionals from other areas were directed. Many healthcare providers suspended the implementation of planned medical procedures, limiting themselves only to necessary procedures, and services provided by general practitioners were in the form of telephone consultations [35,36]. All this meant that national healthcare systems faced completely new challenges that could not remain without affecting the quality of entire systems and the health services they provide.

Taking the above aspects into account, the goal of this study is, therefore, to fill the research gap by examining the impact of crisis in the health system (in the example of the COVID-19 pandemic) on individual outcomes of patient service in healthcare using the example of economically developed countries. The article focuses on the resources available to national health systems from the point of view of their preparedness for crisis phenomena, i.e., pandemics and the ability to deal with them.

## 2. Literature Review

### 2.1. Quality in Healthcare 

The quality of health services is one of the key aspects of the healthcare sector and translates into the functioning of all other sectors. Therefore, more and more attention is paid to it both at the micro and macro levels. Its issues are addressed in various agendas and policies at the national, European, and international levels. The World Health Organization notes in its handbook that each country may have slightly different incentives to improve healthcare quality. The main reasons for interest in this topic include: treating high-quality healthcare as a public good, awareness that improving the availability of health services without their adequate quality will not contribute to the desired health outcomes of the population, cost pressure and striving for greater efficiency and cost-effectiveness in the entire healthcare system, or growing expectations public opinion, media, and civil society [37]. The European Commission, treating quality as an important element of a healthcare system’s operation, considers the extent to which these systems meet their objectives. At the international level, there is also a growing interest in quality in the context of the sustainable development goals, such as ensuring access to high-quality essential healthcare services and access to safe, effective, high-quality, and affordable essential medicines and vaccines. The quality of healthcare systems is also of concern to the World Health Organization, which encourages all countries in various guides and good practices handbooks to create and develop national policies and strategies for quality in health systems and to improve the quality of health services [13].

Due to its immateriality, the quality of medical services depends on healthcare, service process, and interaction between patients and the service provider. Therefore, healthcare quality requires a multidimensional definition that considers various views of healthcare stakeholders, especially as it records disputes between service recipients, medical staff, and decision-makers on the quality of healthcare [38].

However, developing the definition of healthcare quality, being understood as a service provided to the customer, did not lead to adopting a single common and universal formula for understanding this term. The authors of significant publications in this field formulate the many significant definitions applied. One of the authors who first dealt with this issue was Donabedian, who defined the quality of health service as “the application of medical science and technology in a way that maximizes health benefits without increasing risk accordingly” (1980), as cited in [2]. The definition formulated by the Joint Commission on Accreditation of Healthcare Organizations sounds similarly—“quality is the extent to which each service provided to the patient and delivered in accordance with the current state of knowledge increases the likelihood of obtaining the desired outcome and reduces the probability of adverse effects” [39]. Øvretveit defines high-quality care as “providing care which exceeds patient expectations and achieves the highest possible clinical results with available resources”, as cited in [40]. The availability of resources in the context of the quality of medical services is also pinpointed by claiming that “the quality of healthcare is defined as the degree of implementation in relation to the specific standard of interventions, which are known to be safe and have the ability to improve health within the resources available” [21]. One of the definitions of the quality of healthcare formulated by the International Organization for Standardization (ISO) (2005) defines the phenomenon given as “the degree to which a set of inherent characteristics fulfills requirements” [41]. The Institute of Medicine (IOM) defines healthcare quality as the “the degree to which health services for individuals and populations increase the likelihood of desired health outcomes and are consistent”, as cited in [42]. Mosadegrad (2013) defined high-quality healthcare as “consistently delighting the patient by providing efficacious, effective and efficient healthcare services according to the latest clinical guidelines and standards, which meet the patients’ needs and satisfies providers” [16]. On the other hand, Valencia-Arias et al. claim that the quality of health services relates to compliance with a set of established regulations, which implies the excellence of operation and satisfaction of the needs of those in need of such care, and thus an improvement in their health conditions [43].

The highlighted definitions underline that healthcare quality is provided to satisfy customer expectations and patient needs and improve care by qualified professional service providers [2]. To better understand the concept of quality of healthcare, it is also important to know the concepts of health services and medical services and the difference between them. “Health services” can be expressed as all the services provided to protect and improve health, prevent diseases, treat patients to the extent possible, prevent disabilities, provide medical and social rehabilitation services to the disabled and provide people with qualified and long life [44]. They cover emergency, preventative, rehabilitative, long-term, hospital, diagnostic, primary, palliative, and home care [45]. Therefore, the quality of healthcare services is described as the level of healthcare services in improving health outcomes, which could be measured by identifying the patients’ or health professionals’ perceptions [46]. Whereas “Medical service” means any medical treatment or any medical, surgical, diagnostic, chiropractic, dental, hospital, nursing, ambulances, or other related services; drugs, medicine, crutches, prosthetic appliances, braces, and supports; and where necessary, physical restorative services [47]. One of the key differences between the two terms is that medical services emphasize disease treatment and care, while health services also emphasize disease prevention and health promotion [48]. Distinguishing the scope of these concepts can be important for decisions made in the health services sector.

The model of the healthcare system adopted in a given country certainly impacts the quality of services. It largely determines the availability of individual medical services and the cost and financial resources available in the entire system. The following three model solutions in healthcare are currently used in developed countries: The first of them is the model of the National Health Service, in which health services are financed from budgetary funds from taxes. In this system, the state is responsible for providing services (most healthcare entities are in the public sphere), finance, and regulation. It combines the elements of universal social security and public guarantee of access to healthcare. The second model is the social insurance system (social health insurance) based on the obligation of health insurance for employees. They cannot decide whether or not to sign up for health insurance because they are forced to. Services are provided by public healthcare providers, non-profit NGOs, and private for-profit companies. The public sector dominates the hospital sector, while the private sector dominates outpatient care. Funding is based on health insurance premiums only. The third model is the private insurance system, based on voluntary health insurance. It does not provide for the obligation to insure against health risks—citizens are free to choose whether to take out insurance. The insurance policy can be taken out with one of the private insurers, which can be for-profit insurance companies or not-for-profit institutions and funds. This model assumes the dominance of the private sector in healthcare and a limited degree of public regulation. Benefits are financed by insurance institutions, and those who cannot or do not want to be insured pay for the required health services out of their pocket. The first two of the above-mentioned model solutions in OECD countries dominate social health insurance and the National Health Service [49,50,51].

### 2.2. The Dimensions of the Quality of Health Services

Various definitions are closely related to monitoring the quality of healthcare based on different criteria for assessing patient content and satisfaction within the established quality control systems. Against the background of the definition of quality, it becomes important to distinguish its attributes in terms of assessment by the patient as the customer [52].

Health service quality management is an interdisciplinary issue. The concept of quality relates to the correctness of the implementation of a medical service in light of the current knowledge and standards and the factors important to the patient. The subject literature presents their wide range, with the specific sets indicated by individual authors as specific models for measuring the quality of healthcare services. They were distinguished and characterized in detail, among others, by Endeshaw (2021) as cited in [39] or Lee and Kim (2017) in their works [2]. Øvretveit developed a system for improving healthcare quality based on three dimensions of quality: professional, customer, and management quality [40]. Professional quality is based on the opinions of professionals on whether professionally assessed consumer needs were satisfied using appropriate services and at the expected level. On the other hand, the quality of management ensures that services are delivered in a resource-efficient manner [20]. 

Dagger et al., in their studies [53], list an interpersonal dimension (relationships, interactions, and communication between the service provider and the patient), technical (the results of the service process and technical competencies of the service provider), environmental (environmental factors which influence the patient perception), and administrative (enables the implementation of basic services, focusing on timeliness, operation, and support for patients). In turn, Venkatesh et al. indicated technical quality, interpersonal aspects, communication, financial aspects, time spent with a doctor, and accessibility and comfort in assessing the level of patient service [54]. Similarly, Donabedian, as cited in [16], distinguished technical quality (relating to the efficiency of care in generating attainable health benefits), interpersonal quality (concerning the extent of adjusting patient needs and preferences), and facilities (which include the characteristics such as physical comfort of the environment and attributes of service delivery organization). Authors often [39,55,56] refer to three categories determining the quality of the medical service: structure (well-qualified, well-equipped, and well-organized environment), process (adequacy and skills in performed activities), and result, i.e., health-related indicators (e.g., mortality, morbidity, complications, side effects, or patient satisfaction with the treatment).

However, most often, the quality of health services is defined in two dimensions [39,57,58]:

1. technical—from the point of view of the institution providing health services, and
2. functional—from the point of view of the patient.

Technical quality, a prerequisite for functional quality, includes all the elements essential for manufacturing the actual product. Thus, everything that affects the result of operational processes, e.g., medical equipment, equipment, knowledge and professional skills of employees, medical technologies used, procedures and instructions applied, or adopted standards. Technical quality is, however, difficult to assess for patients. Therefore, functional quality, determined by the dimension of contacts between the service provider and the recipient, is growing in importance. The resulting quality aspect includes effective, efficient, fair, timely, safe, and focused patient care, while the interpersonal quality aspect includes exchange of information, kindness, mindfulness and development of understanding, and collaboration through the exchange of information. Functional quality is the result of the patient’s assessment of medical service and is the sum of their experiences in relation to the specific medical entity. However, it should be remembered that patients expect a reliable examination or surgery from medical workers, as should the recognition of values held by the patient of providing understandable information, support, and relief in suffering. Nevertheless, it should be noted that users who do not know the difference between these concepts usually perceive the quality of services from a purely functional perspective, which makes it difficult for them to accurately assess the health benefits received [43]. 

Upadhyai et al. [57] classified the dimensions of the quality of health services characterized above as medical aspects of care. In contrast, they considered the non-medical as those which indirectly influence health and well-being and included three dimensions: servicescape, accessibility, and responsiveness. Servicescape was defined by Booms and Bitner (1981) as “the environment in which the service is created and in which the seller and the customer interact and, in combination with tangible goods, which facilitates the performance or communication of the service”, as cited in [59]. The servicescape of healthcare includes attributes such as infrastructure, institution, food and room, physical environment, and cleanliness. The dimension of accessibility relates to attributes such as financial and physical access, comfort, admission and discharge of the patient, and other administrative processes. Responsiveness, among others, includes patient dignity and autonomy, confidentiality of care, access to the social support network during care, and the quality of basic facilities [57]. Considering a comprehensive view of the quality of health services, distinguishing a large number of factors determining its high level, patient service quality management is increasingly becoming a constant challenge for health organizations since it plays a key role in their development, success, and sustainability [43]. 

During the COVID-19 pandemic, the focus shifted from patients as sole clients of healthcare organizations to strengthen their role as direct strategic partners in the decision-making process [60]. This fits in with the concepts of patient-centered care (PCC) and shared decision making, which are gaining more and more recognition worldwide and, in many countries, are being implemented as standards in medical care [61,62,63,64,65,66,67]. Patient-centered care is defined as care delivery that respects and integrates patients’ wants, needs, and preferences into goal setting and treatment [68]. This concept focuses on treating patients individually, respecting their rights, and building mutual trust and understanding between the patient and the healthcare professional. It incorporates a person’s preferences, values, and beliefs into the decision-making process. It also enables the creation of a treatment plan that is relevant and meaningful to the treated. Thanks to this, the patient is actively involved in his care instead of being its passive recipient [69,70,71]. PCC contributes to improving healthcare quality by combining the technical knowledge of healthcare professionals with the experience of patients [72], and this results in higher satisfaction ratings and better health outcomes [68].

Data from recent years show that health systems were not prepared to manage such a significant change as the pandemic. The efficiency of national systems during a pandemic can be assessed, among other things, by the availability of physical healthcare resources. In order to show the scale of the impact of this type of crisis on the health system, the availability of these resources in a selected period was examined. It was assumed that the level of patient service differed in demographic, financial, human and technical resources, and the scope of available healthcare services before and after the pandemic. Thus, the following hypotheses were framed.

**H1.**  *The results from the first measurement do not differ from those from the second.*

**H2.**  *The results of the first measurement differ from the results of the second.*

## 3. Materials and Methods

### 3.1. Research Samples and the Research Process

In order to take into account the resources of various national health systems in the study, data from European countries were selected for analysis. The group of OECD countries brings together countries, and the primary objective of this international group of countries is, among others, to ensure an adequate standard of living in the member states, which may include high access to healthcare services. This standard also includes ensuring the appropriate quality of access to the health system, and ensuring high-quality patient service is an important priority of the policy of this organization. OECD countries provide about 2/3 of global commodity production, 3/5 of global exports and 4/5 of total public development aid. Therefore, OECD countries and selected non-member economies were selected for the study. The database was downloaded and prepared based on data on healthcare resources available in the OECD statistical database [73]. In total, the sample included 38 countries, i.e., Australia, Austria, Belgium, Canada, Chile, Colombia, Costa Rica, Czech Republic, Denmark, Estonia, Finland, France, Germany, Greece, Hungary, Iceland, Ireland, Israel, Italy, Japan, Korea, Latvia, Lithuania, Luxembourg, Mexico, the Netherlands, New Zealand, Norway, Poland, Portugal, Slovak Republic, Slovenia, Spain, Sweden, Switzerland, Turkey, the United Kingdom, and the United States. Table 1 groups countries by population size and indicates the most important characteristics (average value for countries in a given group) regarding the health system.

Based on population size, OECD countries are grouped into six groups. Table 1 indicates that the population size is not related to the highest value of the studied indicators in a given group of countries. The highest level of average primary care is in countries with a population of between 10 million and 20 million people. The largest current expenditure on health and hospitals is in the countries with the largest population. In turn, countries with a population of less than 10 million have the highest levels of government and compulsory health insurance schemes, out-of-pocket expenditure, and total health and social employment.

To assess whether the pandemic had an impact and in what areas on the level of patient service in healthcare, the differences between the two dependent research groups were tested, i.e.,

(I) the level of availability of healthcare resources before the pandemic—2019 year;
(II) the level of availability of healthcare resources during the pandemic—2020 year.

The research trials differed in the measurement time carried out in 2019 and 2020. This means that the factor that differentiated the measurements was the time factor that differentiated the pairs of dependent variables. 

To verify the main hypothesis, the phenomenon examined was tested using the T-test method for dependent samples, which was used to compare the means of the two measurements, i.e., pretest–posttest. In both measurements, the mean values of the same selected indicators characterizing the level of healthcare in selected countries were analyzed. The obtained results of the T-test provide information on whether the independent variable, i.e., measurement time, differentiates or not the level of dependent variables, i.e., the level of patient service due to the availability of healthcare resources. At the same time, the T-test result is affected by the size of the tested sample, which is small in the case examined. With the same strength of the impact of the independent variable, the value of the T statistics will increase as the sample size increases. Therefore, in the subsequent step, the effect size measure was applied to measure the strength of the relationship between the dependent and independent variables. The use of this measure allowed for comparing the results, which consider the same indicators describing the dependent variable. The commonly used measure of the effect size in the case of the difference between two means is Cohen’s d statistics, but on smaller samples, such as ours, it can bias the results. Therefore, applying Hedge’s g corrects the original effects to produce less biased results [74,75]. 

### 3.2. Variables in the Research

Due to the established research, the period of 2019–2020 was considered. The year of 2019 is used as the base year, i.e., the year before the pandemic. In turn, 2020 is the year of the outbreak of the pandemic in the countries examined, when the first effects of the introduction of sanitary restrictions were recorded. Each of the two research groups were characterized, including the seventeen indicators (also known as quantitative variables) divided into 5 areas, as presented in Table 2. 

The five selected categories of resources are directly or indirectly related to the quality of services provided in the healthcare system. The category of human resources (no. 3) and the category of services (no. 5) are directly related to the services provided. Other categories of resources (no. 1, 2, 4) should be defined as supplementing the quality of patient service in the entire health system.

## 4. Testing Differences in the Area of Demographics, Finance, Resources, and Services of Healthcare

### 4.1. T-Test in the Demographic Area 

**Figure 1:**
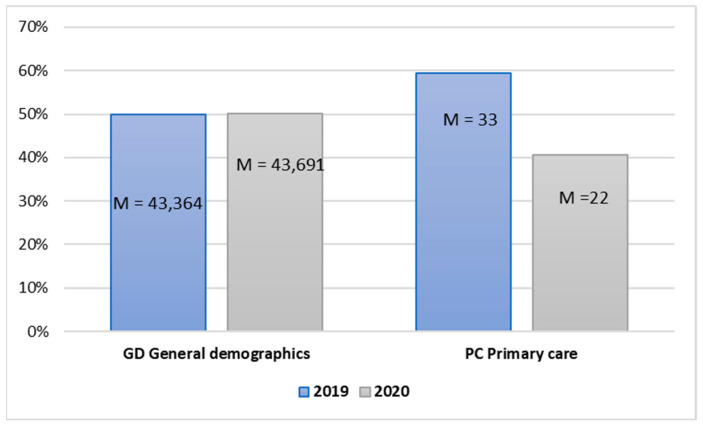
Independent sample mean values in the area of demographic data.

The population size was distinguished in the area of demographic variables, determining the equality of samples in the research in particular years. Moreover, the general issue of the quality of primary care expressed was referred to (Figure 1).

The average population size in 2019 and 2020 was similar—over 43 million people. Comparing the means in the examined years for the subsequent variables is possible due to the similar size of the research sample population. In turn, the primary care indicator expressed as an age–sex standardized rate per 100,000 population was slightly lower in 2020 than in the year before the pandemic. The value *p* < 0.001 for Pairs 1 and 2 indicates significant differences between the means for these variables, meaning the year statistically differentiates significantly their individual values (Table 3). 

The value of the T-test for Pair 1 t(38) = −2.048 and *p* < 0.05, and the test results for Pair 2 t(38) = 6.920 and *p* < 0.001, which indicated the occurrence of significant differences between the examined years, both in the level of the population and the quality of primary care. The mean value for Pair 1 M = −327.77 indicates that, on average, the population in 2019, compared to the year of the pandemic, was lower by this value. In turn, the mean value for Pair 2 at the level of M = 10.60 means a decline in the primary care indicator in 2020 compared to 2019 before the pandemic.

In the case of Pair 1, Hedge’s g value is 0.004 and shows a minimal effect size, which means that the occurrence of the pandemic had a low impact on the changes in the population level in the period examined. In the case of Pair 2, the value of Hedge’s g test = 1.086 indicates a strong relationship between the level of primary healthcare and the outbreak of the COVID-19 pandemic (Table 4).

### 4.2. T-Test in the Financial Area

**Figure 2:**
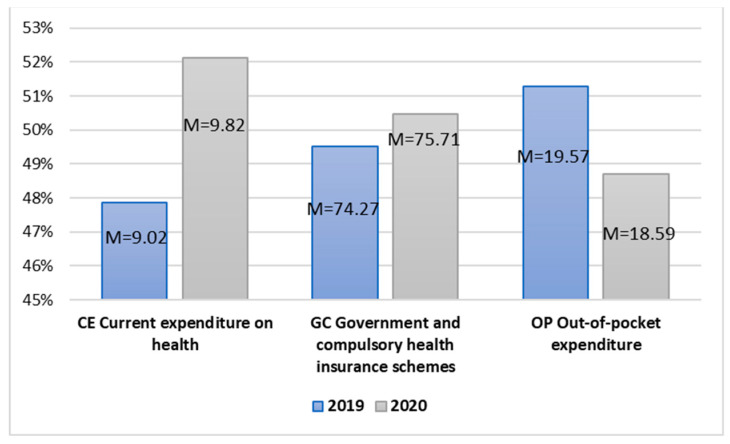
Independent sample mean values in the area of healthcare financing.

The occurrence of the pandemic was related to a clear increase in the need for new healthcare measures, including vaccines and related research aimed at searching for efficient solutions for human life (Figure 2). 

The average current expenditure on healthcare and government and compulsory health insurance schemes in 2020 were higher than in the previous year. In turn, the out-of-pocket expenditure indicator, on the contrary, was higher before the pandemic. This means that government and current expenditures increased during the pandemic. In turn, the out-of-pocket expenditure of natural persons related to care and treatment was reduced. For all the examined pairs of dependent variables (no. 3, 4, 5), the correlation values at the level equal to or higher than 0.988 for *p* < 0.001 mean a significant positive relationship between the examined pairs of variables (Table 5). This means that the differences between the mean values of expenditure on healthcare are statistically significant. 

The T-test values for Pair 3 t(38) = −11.863 and *p* < 0.001 and for Pair 4 t(38) = −7.414 and *p* < 0.001 allow for assuming that the level of current and government expenditure on healthcare was, on average, significantly higher during the pandemic than before its outbreak. In turn, for Pair 3 t(38) = 7.446, the mean of out-of-pocket expenditure was significantly lower during the pandemic. The value of current expenditure was, on average, 0.8031% of gross domestic product higher in 2020 than in the previous year, such as the value of government expenditure, which, during the pandemic, was higher by over 1.43% of current expenditure on health. In turn, out-of-pocket expenditure decreased on average by 0.98% of current expenditure on health in 2020 in relation to 2019. 

In the case of Pair 3, Pair 4, and Pair 5, Hedge’s g value, respectively, amounts to 1.862, 1.164, and 1.169, which is evidence of the considerable scale of the effect. This means that the occurrence of the pandemic had a significant impact on the level of current expenditure care as well as government and out-of-the pocket expenditure on healthcare (Table 6).

### 4.3. T-Test in the Area of Human Resources in Healthcare 

**Figure 3:**
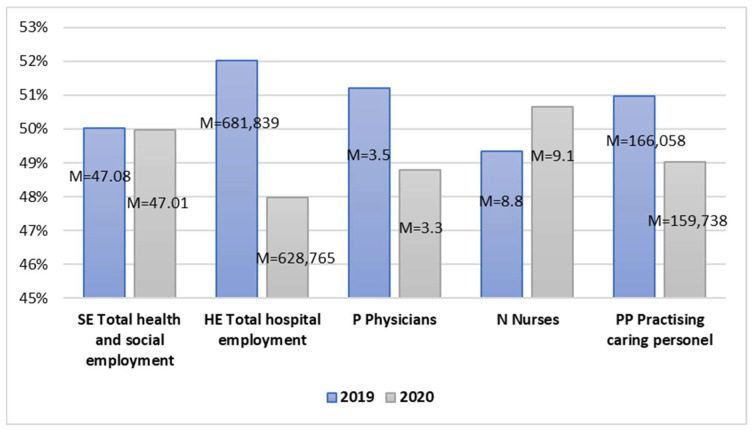
Independent sample mean values in the area of human resources in healthcare.

Human resources available in healthcare played a key role during the fight against the pandemic. An increased level of emergencies resulted in an increased demand for healthcare workers, including primary nurses (Figure 3). 

The average total health and social employment level in the analyzed period was similar in the examined years. In turn, when considering total employment in hospitals, this mean was lower during the pandemic. Broken by the structure of employment, a decline in the number of employees in 2020, in relation to 2019, was recorded in the case of physicians and practicing caring personnel. In turn, at the same time, the average number of nurses employed in healthcare increased. The correlation values for all the pairs of variables are equal and higher than 0.961 for *p* < 0.001, which amounts to a significantly strong and positive relationship (Table 7). This means that the differences between the mean values of all the examined variables in the area of human resources in healthcare are statistically important due to the research period, which was the time before and after the outbreak of the COVID-19 pandemic.

The T-test value for Pair 6 t(38) = 0.283 and *p* = 0.389, Pair 7 t(38) = 1.549 and *p* = 0.065, and Pair 10 t(38) = 0.599 and *p* = 0.276 did not indicate statistically significant differences between the means. In turn, the test value for Pair 8 t(38) = 3.818 and *p* < 0.001 allows for assuming that the average number of physicians significantly differs in the analyzed period. In 2020 the number of physicians was significantly lower than in the previous year. The test value for Pair 9 t(38) = −5.445 and *p* < 0.001 indicates that the average number of nurses was significantly higher during the pandemic. For the significant pairs of dependent variables, Hedge’s g test was conducted. The results are shown in Table 8.

In the case of Pair 8, d = 0.599 means a moderate effect size. In turn, Pair 9, d = 0.855, amounts to the large effect of the impact. Therefore, one may assume that the occurrence of the COVID-19 pandemic significantly impacted the number of physicians employed during the pandemic and nurses even more (Table 8).

### 4.4. T-Test in the Area of Technical Resources 

**Figure 4:**
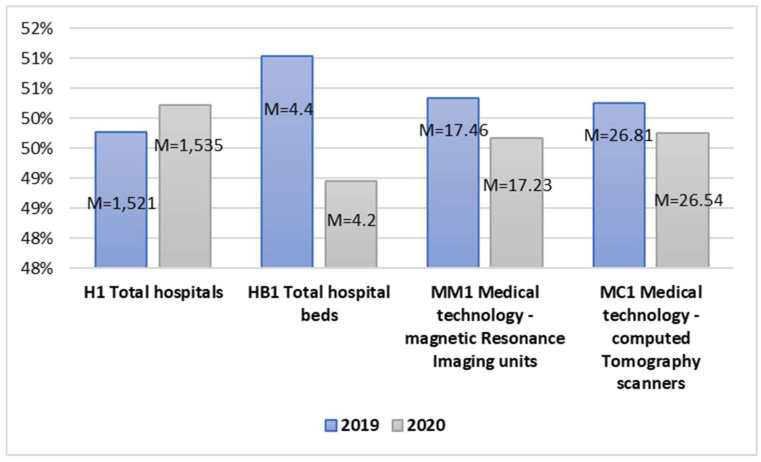
Independent sample mean values in the area of technical equipment in healthcare.

Ensuring the adequate quality of patient service during the pandemic was associated with numerous restrictions and the related access to hospitals, hospital beds, or tests not directly linked to COVID-19 (Figure 4). 

In the analyzed period, the average number of hospitals increased in 2020 in relation to 2019. The other variables in the area of technical resources declined during the pandemic. The number of beds available for patients per 1000 population decreased. In addition, during the COVID-19 pandemic, the number of medical procedures performed using medical equipment, i.e., magnetic resonance and computed tomography scanners, also declined. Testing the significance of the differences between the means for the examined variables allowed for the observation that the correlation values for all the pairs of variables are at the level r equal and higher than 0.964 for *p* < 0.001, which means a strong and positive relationship (Table 9). This means that the differences between the mean values of all the examined variables in the area of technical resources in healthcare are statistically significant, broken down by the analyzed period before and after the outbreak of the COVID-19 pandemic.

The T-test value for Pair 11 t(38) = −1.587 and *p* = 0.060, Pair 13 t(38) = 0.954 and *p* = 0.173, and Pair 14 t(38) = 0.317 and *p* = 0.376 did not indicate significant statistical differences between the means. In turn, the test value for Pair 12 t(38) = 0.317 and *p* < 0.001 indicated that the average number of total hospital beds significantly differed in the examined period and was lower during the pandemic than before. 

Pair 12 Hedge’s g value is 1.063, indicating a huge effect size. Therefore, the outbreak of the pandemic had a significant effect on the number of available hospital beds at that time (Table 10). 

### 4.5. T-Test in the Area of Care Services in Healthcare

**Figure 5:**
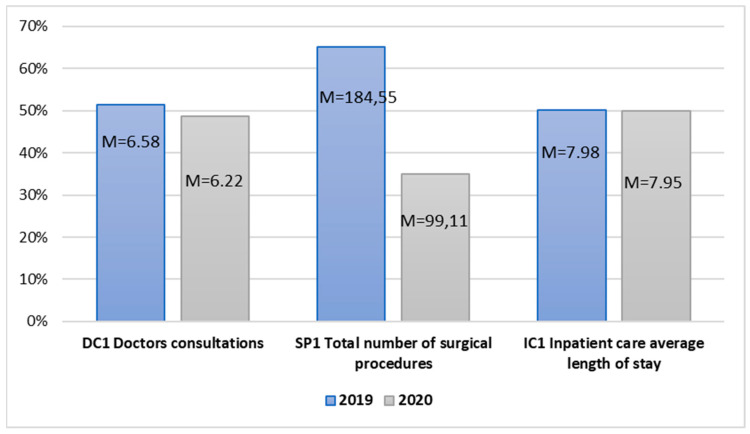
Independent sample mean values in the area of care services in healthcare.

The last area examined includes the services dedicated to patients in health centers and hospitals. Doctor consultations, surgical procedures, and inpatient care average length of stay were considered (Figure 5). 

The mean values of the examined variables in the area of healthcare organization in 2019–2020 allow for the observation that all these variables declined during the pandemic outbreak. The average length of stay of the number of provided doctor consultations and inpatient care decreased. The average number of all performed surgical procedures dropped significantly in 2020. The T-test results for dependent samples indicate, for each analyzed pair of variables, the correlation values at the level r equal and higher than 0.990 for *p* < 0.001 (Table 11). This means that the value of each correlation is very high and positive and indicates significant differences between the means for the analyzed pairs of variables. 

The T-test value for Pair 17 t(38) = 0.358 and *p* = 0.361 did not indicate significant statistical differences between the means for the variable concerning inpatient care average length of stay. In turn, in the case of Pair 15 t(38) = 7.401 and *p* < 0.001 and Pair 16 t(38) = 4.180 and *p* < 0.001, it was observed that the average number of doctor consultations and performed surgical procedures was significantly lower during the pandemic than before its occurrence. 

In the case of Pair 15, Hedge’s g value amounts to 1.185 and indicates a strong effect size, which means that the outbreak of the pandemic had a large impact on a reduced number of doctor consultations during the pandemic. In turn, Pair 16 Hedge’s g test value = 0.678 indicates a moderate pandemic effect in the case of the decline in the number of performed surgical procedures (Table 12). 

## 5. Summary and Discussion of the Research Results 

The present research selected healthcare resources, including the period before and during the COVID-19 pandemic, as an example of crisis in the health system. The data from several countries allowed for some observations that partially confirm the adopted research hypotheses that the level of patient service, conditioned by the availability of resources, significantly differs due to the occurrence of the pandemic. A similar study, comparing the periods before and at the peak of the pandemic, was carried out by Moynihan et al. [76]. The criteria of their survey assessment included, among others, visits, admissions or hospitalizations, diagnostic services, and therapeutic and preventive interventions. However, these studies were based on a literature review and did not cover the full years 2019–2020. In turn, Ivanov et al. [77] conducted the research over several months of the pandemic and concentrated their research efforts on improving the patient service quality. In addition, they focused on nine hospitals in Serbia, so their research is regional in scope. Okeke [78], in his research based on routine visits to primary care clinics in Nigeria, proved that the quality of healthcare interactions decreased significantly in the early months of the pandemic. Again, however, these were territorially and time-limited studies. Pereira et al. [79] showed that the COVID-19 outbreak (March and April 2020) was associated with a significant reduction in hospital admissions for ACS and STEMI, as well as a reduction in PPCI. Bruch et al. [33] analyzed the consequences of the COVID-19 pandemic on outpatient care in Brandenburg between 22 March and 4 May 2020. They focused on the burden for physicians and psychotherapists in outpatient practices and alternative ways to provide care, particularly telehealth. The results of their research indicate that almost all physicians and psychotherapists recorded fewer admissions, while the number of teleconsultations increased significantly. A significant limitation of both studies is the short time horizon. However, the present study included the analysis of statistical data, which indicated that the quality of patient service is significantly different in the area of the indicators of demographics, finance, human resources and technical resources, and the scope of available services. As a result, the researchers distinguished measures supporting activities aimed at improving the quality of healthcare during the crisis in the health system.

Table 13 contains the research results and their impact on patient service during and before the pandemic. Some of them did not indicate significant differences in the analyzed period, and the examination of the impact effect did not indicate that these differences resulted from the occurrence of the pandemic. 

The comparison of the average value of the indicator for the analyzed years allowed for indicating that the level of primary healthcare during the pandemic is significantly statistically lower than in the conditions before the pandemic. This means that the null hypothesis should be rejected in favor of the alternative hypothesis. The pandemic resulted in increased current and government expenditures and a reduction in out-of-pocket expenditures on healthcare due to the pandemic outbreak. Moreover, the pandemic significantly impacted a decline in the number of employed physicians and an increase in the employment of nurses in healthcare. This is confirmed by the conclusions from the ASPE report [80], according to which the pandemic greatly impacted healthcare professionals, leading to labor shortages. Both professional groups were seriously exposed to illness, burnout, or death risks. At the same time, during the pandemic, the number of hospital beds dropped significantly. The Italian healthcare study conducted by Giancotti [81], covering several months of 2020, shows that the supply of health services and public hospitals, including technical background, was not sufficiently prepared. Additionally, Candel et al. [82] analyzed the problem of no beds in building temporary hospitals as an example of flexibility and adaptation in an epidemic. The presence of COVID-19 also had a large impact on reducing the number of doctor consultations and performed surgical procedures at that time. The literature research results by Moynihan et al. [76] also confirm the deterioration of medical services in general during the pandemic, whereas, according to their analyses, the highest decrease was related to medical visits, diagnostics, and admissions. 

The research results are, therefore, generally consistent with the existing results in the literature. However, their advantage is a more comprehensive approach to the impact of crisis events, such as the COVID-19 pandemic, on the quality of healthcare and the fact that data from many countries were analyzed. Therefore, they are not burdened with regional limitations resulting, for example, from the introduction of specific restrictions and methods of fighting the pandemic. In addition, they indicate the differences between the pre-pandemic period and during the pandemic using data for whole years.

## 6. Conclusions

The quality of healthcare services directly affects the life of individuals and society. The crisis caused by the pandemic influenced this quality, constituting a significant barrier that prevents effective patient care. Undoubtedly, the key strategy for the survival of national public health systems is to satisfy the needs and expectations of patients regardless of the changes taking place and the conditions of their functioning. To this end, it becomes important to identify the areas of healthcare most susceptible to these changes in order to predict and plan actions that satisfy the needs of healthcare at the highest possible level. 

The conducted research confirmed that the level of patient service significantly differs due to the occurrence of the pandemic, which is of the nature of sudden and rapid changes in the healthcare system, primarily resulting in the deterioration of the quality of healthcare services. To ensure at least the existing and higher level of service in the period of high morbidity of society, the focus should be placed on the areas that deteriorated significantly during the COVID-19 period. In turn, the pandemic itself should be treated as a test of the national healthcare system and an opportunity to improve it. It was indicated that despite an increase in some indicators of patient service during the pandemic, most of them deteriorated. To improve the healthcare system, it is primarily necessary to ensure the continuity of operational procedures, employ adequate staff, and increase access to medical consultations. At the same time, the conducted study has some limitations that should be considered in further research. The analyzed data come from the period of the pandemic’s beginning and include only selected aspects. In the future, the research period should be extended to before, during, and after the pandemic, comparing the present results to those. Moreover, the scope of the analyzed phenomenon may be considered, considering mental health factors as important during the pandemic period. The currently obtained results can be treated as a determinant for planning and organizing patient healthcare in the event of an epidemic, pandemic, or other similar phenomena affecting human health and life.

The study has some limitations. First, only OECD countries were considered, while the pandemic had a global scope. In addition, due to institutional and cultural settings, individual countries’ health systems differ from each other, which may affect the differences in the results obtained. At the same time, it can be the background for further research in this area, which can be supplemented with research among hospital patients during the pandemic. 

## Figures and Tables

**Table 1:** Average value of the essential characteristics of the health system in OECD countries.

	General Demographics	Average of Primary care (Age-Sex Standardised Rate Per 1 mln Population)	Current Expenditure on Health (% of Gross Domestic Product)	Government and Compulsory Health Insurance Schemes, % of Current Expenditure on Health	Out-Of-Pocket Expenditure (% of Current Expenditure on Health)	Total Health and Social Employment (Density Per 1000 Population)	Hospitals Number (in Thousand)
United States; Mexico; Japan	More than 100 mln	17	12	73	21	38	6.4
Germany; Turkey; France; United Kingdom; Italy; Korea, Colombia; Spain; Poland; Canada; Australia	More than 20 mln	29	10	76	17	43	2.6
Chile; Netherlands; Belgium; Greece; Czech Republic; Sweden; Portugal	More than 10 mln	15	10	74	21	43	0.3
Hungary; Israel; Austria; Switzerland; Denmark; Finland; Slovak Republic; Norway; Costa Rica; New Zealand	More than 5 mln	22	10	76	18	56	0.1
Ireland; Lithuania; Slovenia; Latvia; Estonia	More than 1 mln	28	8	72	22	36	0.05
Luxembourg; Iceland	Less than 1 mln	6	8	85	12	75	0.009

**Table 2:** Characteristics of the set of variables.

Name of the Data Area	Full Name of the Variable	Measurement Unit	Indication of the Variable in the Database	
			2019	2020
1. Demographic data	General demographics	Thousands of persons	GD1	GD2
	Primary care	Age–sex standardised rate per 100,000 population	PC1	PC2
2. Financial data	Current expenditure on health	% of gross domestic product	CE1	CE2
	Government and compulsory health insurance schemes	% of current expenditure on health	GC1	GC2
	Out-of-pocket expenditure	% of current expenditure on health	OP1	OP2
3. Data—human resources	Total health and social employment	Density per 1000 population (head counts)	SE1	SE2
	Total hospital employment	Density per 1000 population (head counts)	HE1	HE2
	Physicians	Density per 1000 population (head counts)	P1	P2
	Nurses	Density per 1000 population (head counts)	N1	N2
	Practising caring personnel	GDNumber of persons (head counts)	PP1	PP2
4. Data—technical resources	Total hospitals	Number of hospitals	H1	H2
	Total hospital beds	Per 1000 population	HB1	HB2
	Medical technology-magnetic Resonance imaging units	Total, Per million population	MM1	MM2
	Medical technology—computed Tomography scanners	Total, Per million population	MC1	MC2
5. Service data	Doctors consultations	Number per capita	DC1	DC2
	Total number of surgical procedures	Number of procedures	SP1	SP2
	Inpatient care average length of stay	Days	IC1	IC2

**Table 3:** Test for dependent samples.

	Differences in Dependent Samples					t	df	*p*
	M *	S.D.	S.E.	95% CI				
				Lower Limit	Upper Limit			
Pair 1	−327.77	999.64	160.07	−651.82	−3.73	−2.048	38	0.024
Pair 2	10.60	9.56	1.53	7.50	13.70	6.920	38	<0.001

* The negative mean amounts to an increase in the mean value of the examined explanatory variable compared to the base measurement and opposite.

**Table 4:** Effect sizes for Hedge’s g dependent samples.

		g Value	95% CI	
			Lower Limit	Upper Limit
Pair 1	GD1 General demographics—GD2 General demographics	0.004	0.318	0.310
Pair 2	PC1 Primary care—PC 2 Primary care	1.086	0.703	1.504

**Table 5:** Test for dependent samples.

	Differences in Dependent Samples					t	df	*p*
	M	S.D.	S.E.	95% CI				
				Lower Limit	Upper Limit			
Pair 3	−0.803	0.422	0.067	−0.940	−0.666	−11.863	38	<0.001
Pair 4	−1.434	1.208	0.193	−1.825	−1.042	−7.414	38	<0.001
Pair 5	0.982	0.823	0.131	0.715	1.249	7.446	38	<0.001

**Table 6:** Effect sizes for Hedge’s g dependent samples.

		g Value	95% CI	
			Lower Limit	Upper Limit
Pair 3	CE1 Current expenditure on health and CE2 Current expenditure on health	1.862	2.424	1.366
Pair 4	GC1 Government and compulsory health insurance schemes and GC2 Government and compulsory health insurance schemes	1.164	1.594	0.771
Pair 5	OP1 Out-of-pocket expenditure and OP2 Out-of-pocket expenditure	1.169	0.776	1.600

**Table 7:** Test for dependent samples.

	Differences in Dependent Samples					t	df	*p*
	M	S.D.	S.E.	95% CI				
				Lower Limit	Upper Limit			
Pair 6	0.067	1.48602	0.23795	−0.41427	0.54915	0.283	38	0.389
Pair 7	53,073.51	213,917.73	34,254.25	−16,270.59	122,417.61	1.549	38	0.065
Pair 8	0.166	0.273	0.043	0.078	0.255	3.818	38	<0.001
Pair 9	−0.241	0.276	0.044	−0.330	−0.151	−5.445	38	<0.001
Pair 10	6320.05	65,902.62	10,552.86	−15,043.10	27,683.21	0.599	38	0.276

**Table 8:** Effect sizes for Hedge’s g dependent samples.

		g Value	95% CI	
			Lower Limit	Upper Limit
Pair 8	P1 Physicians and P2 Physicians	0.599	0.266	0.950
Pair 9	N1 Nurses and N2 Nurses	0.855	1.237	0.498

**Table 9:** Test for dependent samples.

	Differences in Dependent Samples					t	df	*p*
	M	S.D.	S.E.	95% CI				
				Lower Limit	Upper Limit			
Pair 11	−13.530	53.272	8.530	−30.807	3.730	−1.587	38	0.060
Pair 12	0.180	0.166	0.027	0.126	0.234	6.772	38	<0.001
Pair 13	0.232	1.518	0.243	−0.260	0.724	0.954	38	0.173
Pair 14	0.268	5.287	0.846	−1.445	1.982	0.317	38	0.376

**Table 10:** Effect sizes for Hedge’s g dependent samples.

		g Value	95% CI	
			Lower Limit	Upper Limit
Pair 12	HB1 Total hospital beds and HB2 Total hospital beds	1.063	0.683	1.477

**Table 11:** Test for dependent samples.

	Differences in Dependent Samples					t	df	*p*
	M	S.D.	S.E.	95% CI				
				Lower Limit	Upper Limit			
Pair 15	0.357	0.302	0.048	0.260	0.455	7.401	38	<0.001
Pair 16	85,444.07	125,997.14	20,439.43	44,029.84	126,858.30	4.180	37	<0.001
Pair 17	0.031	0.543	0.088	−0.147	0.210	0.358	37	0.361

**Table 12:** Effect sizes for Hedge’s g dependent samples.

		g Value	95% CI	
			Lower Limit	Upper Limit
Pair 15	DC1 Doctors consultations and DC2 Doctors consultations	1.185	0.769	1.592
Pair 16	SP1 Total number of surgical procedures and SP2 Total number of surgical procedures	0.678	0.321	1.028

**Table 13:** Indicators of the level of patient service during the pandemic.

Name of the Data Area	Full Name of the Variable	Statistically Significantly Depends on the Pandemic	Assessment of the Level of Patient Service in 2020 (“+1” an Increase in the Service Level, “–1”—A Decline in the Service Level, “0”—No Impact)
1. Demographic data	General demographics	No	0
	Primary care	Yes	−1
2. Financial data	Current expenditure on health	Yes	+1
	Government and compulsory health insurance schemes	Yes	+1
	Out-of-pocket expenditure	Yes	−1
3. Data—human resources	Total health and social employment	No	0
	Total hospital employment	No	0
	Physicians	Yes	−1
	Nurses	Yes	+1
	Practising caring personel	No	0
4. Data—technical resources	Total hospitals	No	0
	Total hospital beds	Yes	−1
	Medical technology-magnetic Resonance imaging units	No	0
	Medical technology—computed Tomography scanners	No	0
5. Data—service area	Doctors consultations	Yes	−1
	Total number of surgical procedures	Yes	−1
	Inpatient care average length of stay	No	0

## Data Availability

The data was downloaded and selected from the OECD.Stat public database.
